# Evaluating plasma biomarkers NfL, GFAP, GDF15, and FGF21 as indicators of disease severity in Charcot–Marie Tooth patients

**DOI:** 10.3389/fneur.2024.1490024

**Published:** 2025-01-15

**Authors:** Dace Pretkalnina, Elizabete Kenina, Linda Gailite, Dmitrijs Rots, Kaj Blennow, Henrik Zetterberg, Viktorija Kenina

**Affiliations:** ^1^Department of Neurology and Neurosurgery, Children’s Clinical University Hospital, Riga, Latvia; ^2^Department of Doctoral Studies, Riga Stradins University, Riga, Latvia; ^3^14th European Reference Network in Neuromuscular Disorders (EURO-NMD), Scientific Laboratory of Molecular Genetics, Riga Stradins University, Riga, Latvia; ^4^Faculty of Medicine, Riga Stradins University, Riga, Latvia; ^5^Department of Human Genetics, Radboudumc, Nijmegen, Netherlands; ^6^Department of Psychiatry and Neurochemistry, Institute of Neuroscience and Physiology, The Sahlgrenska Academy at the University of Gothenburg, Mölndal, Sweden; ^7^Clinical Neurochemistry Laboratory, Sahlgrenska University Hospital, Mölndal, Sweden; ^8^Department of Neurodegenerative Disease, UCL Institute of Neurology, London, United Kingdom; ^9^UK Dementia Research Institute at UCL, London, United Kingdom; ^10^Hong Kong Center for Neurodegenerative Diseases, Hong Kong, China; ^11^Wisconsin Alzheimer’s Disease Research Center, University of Wisconsin School of Medicine and Public Health, University of Wisconsin-Madison, Madison, WI, United States; ^12^Department of Biology and Microbiology, Riga Stradins University, Riga, Latvia; ^13^Rare Neurological Disease Centre, Pauls Stradiņš Clinical University Hospital, Riga, Latvia

**Keywords:** CMT, biomarker, NFL, FGF21, GFAP - glial fibrillary acidic protein, GDF15

## Abstract

**Background:**

Charcot–Marie–Tooth disease (CMT), a slowly advancing hereditary nerve disorder, presents a significant challenge in the medical field. Effective drugs for treatment are lacking, and we struggle to find sensitive markers to track the disease’s severity and progression. In this study, our objective was to investigate the levels of neurofilament light chain (NfL), glial fibrillary acid protein (GFAP), fibroblast growth factor 21 (FGF-21) and growth differentiation factor 15 (GDF-15) in individuals with CMT and to compare them to a control group. Our primary goal is to determine whether these biomarker levels are related to the severity of the disease.

**Methods:**

Initially, 44 patients with CMT and 44 controls participated in this study. CMT diagnosis was approved by genetic testing. Disease severity was assessed through clinical evaluations using the CMT Neuropathy Score version 2 (CMTNSv2). NfL and GFAP concentrations were measured using Single molecule array, while FGF-21 and GDF-15 concentrations were measured by enzyme-linked immunosorbent assays.

**Results:**

In the group of patients with CMT, the concentrations of GDF15, FGF21, NfL, and GFAP were significantly higher than in the control group (*p* < 0.05). NfL and GFAP levels were correlated with the CMTNSv2 score (rs = 0.46, *p* = 0.002; rs = 0.31, *p* = 0.04).

**Conclusion:**

Our study has provided confirmation that plasma concentrations of NfL, GFAP, GDF15, and FGF21 are significantly elevated in patients with CMT compared to controls. Furthermore, NfL and GFAP levels were correlated with the clinical severity of CMT. These findings suggest that NfL and GFAP can be reliable disease indicators in future research.

## Introduction

Charcot–Marie–Tooth disease (CMT) represents the most prevalent form of inherited neuropathy. Monitoring its progression and assessing the severity of the condition requires the identification of potential molecular markers (1). Some potential biomarkers are neurofilament light chain (NfL) and glial fibrillary acid protein (GFAP), these are suggested as biomarkers to assess nerve damage and fibroblast growth factor 21 (FGF-21), growth differentiation factor 15 (GDF-15) has been proposed as a biomarker to reduce muscle mass in CMT disease (2–5).

CMT is usually classified based on the type of peripheral neuropathy, respectively,demyelinating or axonal. This classification clinically is measured by the median motor nerve conduction velocity (MNCV) measured during electroneurography. CMT is divided into demyelinating (CMT1), which has an MNCV of <38 m/s; axonal (CMT2), which has an MNCV of >38 m/s; and results in between these velocities are referred to as intermediate. CMT, which has an MNCV of 25 to 45 m/s (6, 7).

NfL is a cytoskeletal protein found in both the central and peripheral nervous systems that gives shape and structure to the neuron. A particularly high concentration of neurofilament is found in neuronal axons, and its levels in blood and/or cerebrospinal fluid are elevated after axonal injury (8). Plasma concentration of neurofilament light chain is a potentially promising biomarker for diseases with axonal degeneration (9, 10). Several studies have described the correlation of plasma neurofilament light chain concentration with the severity of CMT disease (2, 3, 11).

GFAP is a structural protein of glial astrocytes found in astrocytes in the central nervous system, non-myelinated Schwann cells (SCs) in the peripheral nervous system, and intestinal glial cells (12). During nerve degeneration, SCs dissociate from axons and subsequently release GFAP into the serum. Studies have indicated that patients with chronic neuropathy exhibit elevated serum GFAP levels compared to control subjects. Furthermore, a correlation has been observed between GFAP concentrations and functional scores of neuropathy, suggesting that GFAP may serve as an indirect marker of axonal damage (13).

In CMT muscle denervation leads to atrophy and fat accumulation in muscles. Currently, there are no serum-based markers to monitor these changes in CMT. However, there are sensitive and specific quantitative biomarkers for muscle pathology in mitochondrial diseases: FGF21 and GDF15 (4, 14). Studies have suggested that FGF21 and GDF15 may play a pathophysiological role in promoting muscle atrophy (4, 5).

Although no studies have specifically investigated GFAP, FGF21 and GDF15 in CMT, its potential as a biomarker to assess disease severity in CMT is promising. Our research aims to analyze the levels of NfL, GFAP, FGF-21, GDF-15 in both CMT patients and control subjects to determine the feasibility of using these biomarkers as an indicator of the severity of CMT and to assess the reliability of these biomarkers for future studies.

## Methods

In this study, a cohort comprising 44 CMT patients and an equal number of control subjects underwent a comprehensive evaluation utilizing established diagnostic procedures for CMT patients. CMT diagnosis was approved by genetic testing. The evaluation involved a nerve conduction study, which was carried out by a certified specialist following the standard polyneuropathy protocol. Patients with CNS abnormalities were excluded. MRI examinations were conducted for almost all participants to confirm the absence of CNS abnormalities.CMT patients was classified according to nerve conduction velocity (NCV): type1(demyelination) less than 35 m/s and type2 (axon) more than 45 m/s (6). Additionally, disease severity scoring was performed according to the CMT Neuropathy Score version 2 (CMTNSv2). The CMTNSv2 is a validated clinical scoring system designed to evaluate the functional and neurological impact of CMT consistently and quantifiable. It is a composite measure incorporating clinical, electrophysiological, and patient-reported outcomes. The scale has nine items divided into three domains. The first domain is patient-reported sensory and motor symptoms. The second domain is the clinical evaluation of pinprick sensation, vibration sensation and strength in the upper and lower limbs using standardized scale. The third domain is based on electrophysiology ulnar MNCV and radial sensory action potential. Each item is scored on a scale of 0 to 4, where higher scores indicate more significant impairment. The total score ranges from 0 to 36, categorized as mild (1–10), moderate (11–20) and severe (>20) (15).

Blood sampling and subsequent storage procedures adhered to rigorous standard operating protocols. Briefly, certified medical personnel collected blood samples from both patients and control individuals in an outpatient setting. These samples were processed within an hour after collection. Blood samples were collected in tubes containing EDTA and subsequently centrifuged at 20°C and 3,500 rpm for a duration of 10 min. The plasma was then carefully aliquoted and stored at a temperature of −20°C.

The control group in our study consisted of people who exhibited good health without a history of neurological diseases or neurological symptoms.

Plasma neurofilament light chain (NfL) concentration was measured in a similar laboratory setting using the same NfL quantification method as previously described (3). Glial fibrillary acid protein (GFAP) was measured with a Single molecule array (Simoa) assay (Quanterix, Billerica, MA). Fibroblast growth factor 21 (FGF-21) and growth differentiation factor 15 (GDF-15) concentrations were measured using enzyme-linked immunosorbent assays according to instructions from the manufacturer (R&D Systems, Minneapolis, MN). All measurements were performed in one round of experiments using one batch of reagents by board-certified laboratory technicians who were blinded to clinical data. Intra-assay coefficients of variation, determined using internal control samples, were below 10%. Quantikine ELISA assays from R&D Systems were used for the analysis in this study.

All samples were analyzed as singlicates in a single round of experiments using one batch of reagents. Analytical variation was monitored by running quality control (QC) samples at the beginning and end of each plate. Intra-assay coefficient of variation (CV) was calculated from these measurements, as all analyses were completed in a single batch. Dilution factors for the samples followed the manufacturer’s recommendations and were consistent across all experiments.

### Statistical analysis

To evaluate the normality of continuous data, we employed various methods, including histograms, Q-Q plots, and the Shapiro–Wilk test. The Mann–Whitney U test was utilized for nonnormally distributed data. Discrete data were subjected to analysis using Pearson’s chi-square test. Correlations among continuous data were evaluated using the Spearman correlation coefficient. Statistical data analysis was performed with Jamovi (v.2.3). Results were considered statistically significant when the *p* value <0.05.

## Results

Initially, 44 CMT patients and 44 healthy subjects were recruited in this study. The group of CMT patients was stratified into subgroups, namely demyelinating CMT and axonal CMT, based on the findings of nerve conduction studies. There were no significant difference in sex (χ^2^ = 0.0455, *p* = 0.831) or age (*p* = 0.95) between the CMT and control groups. The key characteristics of the study group are summarized in Table 1.

**Table 1:** Baseline characteristics of CMT patients and control group.

Study participants	Number of patients (male/female)	Mean age (SD)	Mean disese duration (SD)	Median CMTNSv2 (IQR)
All CMT patients	44 (22/22)	39.1 (± 19.1)	20.8 (± 14.4)	12 (9.0)
Demyelinating (type1)	31 (15/16)	39.8 (± 18.1)	20.9 (± 14.8)	13 (11)
Axonal form (type2)	13 (6/7)	37.4 (± 21.8)	20.5 (± 14.2)	9 (7)
Control group	44 21/23	39 (± 16.2)	NA	NA

CMT, Charcot–Marie–Tooth disease; SD, standard deviation; IQR, interquartile range. CMTNSv2, CMT Neuropathy Score v.2; NA, not applicable.

Genetic testing was done on all patients, and the diagnosis was confirmed. Mutations found in the CMT1 patient group were PMP22 duplication in 54.5% (*n* = 24), GBJ in 13% (*n* = 6) and MPZ in one case. The mutations found in the CMT2 patient group were AARS1 in 6.8% (*n* = 3), HINT1, and MFN2 in 6.8% (*n* = 3) cases. HSPB1 was found in 4.5% (*n* = 2) of patients. MORC2 was found in one patient, and VRK1 was also found.

**Figure 1:**
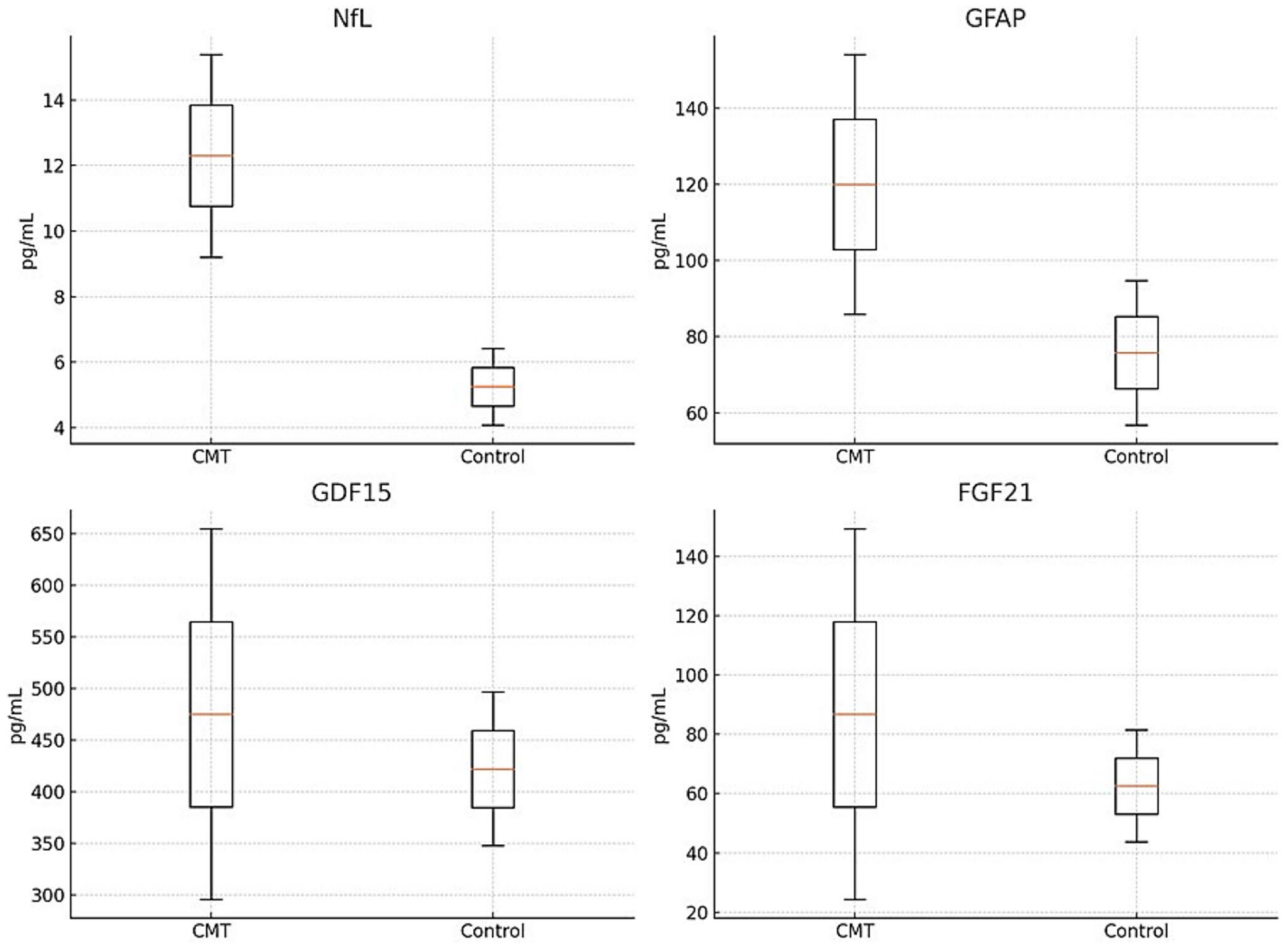
Comparison of NfL, GFAP, GDF15 and FGF21 in CMT and control group.

Consistent with findings from previous studies, our research also confirms that the concentration of NfL in the plasma is higher in the CMT group compared to the control group. Furthermore, the levels of GFAP, GDF15, and FGF21 were also significantly higher in the CMT group than the controls. The precise measurements of these biomarkers in the study groups are detailed in Table 2 and Figure 1.

**Table 2:** Plasma concentrations of NfL, GFAP, GDF15 and FGF21 in CMT and control group.

Study participants	CMT group	Control group	Difference between groups (Mann–Whitney U test)
Median NfL, pg./mL	12.3 (IQR = 6.18)	5.25 (IQR = 2.33)	U = 208 (*p* < 0.001)
Median GFAP, pg./mL	120 (IQR = 68.3)	75.8 (IQR = 37.9)	U = 329 (*p* < 0.001)
Median GDF15, pg./mL	475 (IQR = 359)	422 (IQR = 149)	U = 716 (*p* = 0.035)
Median FGF21, pg./mL	86.8 (IQR = 125)	62.6 (IQR = 37.7)	U = 640 (*p* = 0.006)

CMT, Charcot–Marie–Tooth disease; IQR, interquartile range.

**Figure 2:**
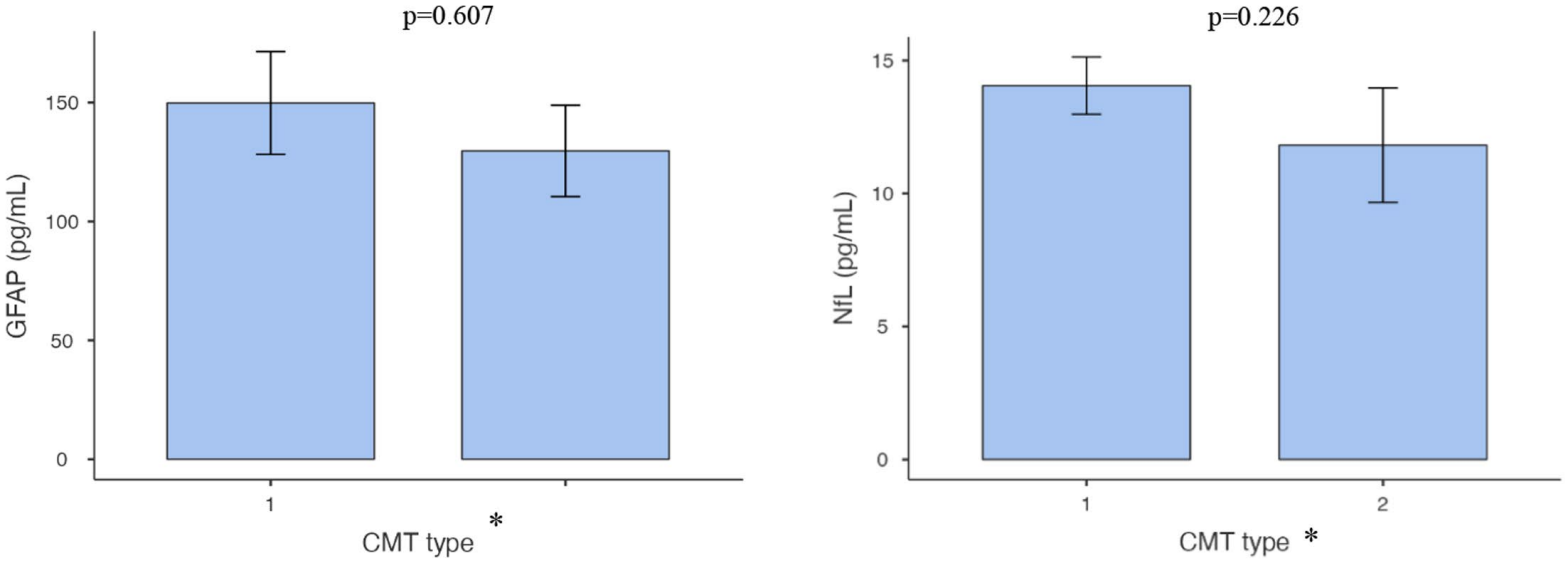
Comparison of NFL and GFAP concentrations in demyelinating vs. axonal CMT groups.

As discussed earlier, some of the biomarkers we have investigated are linked to axonal damage. During nerve degeneration, Schwann cells detach from the axons, releasing GFAP into the serum (16). As well as NfL is closely linked to axonal damage and neuron death (15). Therefore, we compared the median concentrations of these two markers between the demyelinating and axonal subgroups of CMT. However, our analysis did not reveal statistically significant differences in concentration levels between these two subgroups (*p* > 0.05; Figure 2).

To evaluate the relationships between NfL, GFAP, GDF15, and FGF21 levels and various demographic and clinical factors in individuals diagnosed with CMT, encompassing parameters such as age, sex, duration of disease symptoms, CMT subtype, and disease severity, we present the following findings. NfL concentration demonstrated a substantial correlation with the CMTNS (r_s_ = 0.47, *p* = 0.001), highlighting a meaningful association between NfL levels and the severity of the disease. Additionally a notable correlation was observed between NfL concentration and patient age (r_s_ = 0.38, *p* = 0.011), consistent with previous findings indicating that NfL levels tend to increase with age. On the contrary, the concentration of GFAP showed a significant correlation with the duration of the disease symptoms (r_s_ = 0.31, *p* = 0.041) and with CMTNS (r_s_ = 0.31, *p* = 0.043), suggesting a positive relationship between GFAP levels and the severity of the disease.

**Figure 3:**
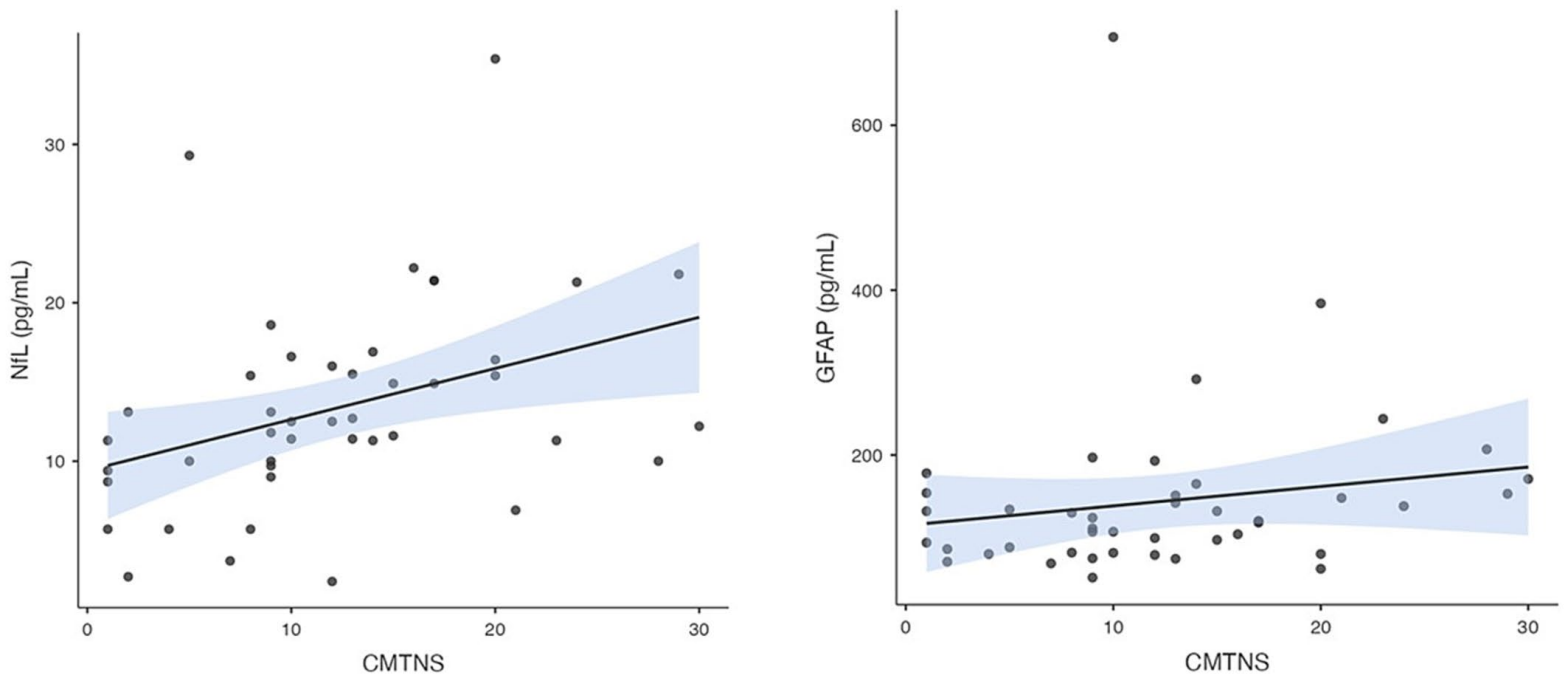
Correlation between plasma NfL and GFAP concentration and CMTNSv2.

GDF15 levels were found to be significantly correlated with patient age (r_s_ = 0.56, *p* < 0.001), and disease duration (r_s_ = 0.37, *p* = 0.015). In contrast to the other biomarkers, FGF21 did not exhibit significant correlations with any of the factors examined. The correlation between disease severity and NfL and GFAP is depicted in Figure 3.

## Discussion

In this study, we provide data on plasma concentrations of NfL, GFAP, GDF15, and FGF21 in individuals with CMT, as well as in a control group. Our analysis revealed that patients with CMT exhibit significantly higher concentrations of these biomarkers than control subjects, suggesting their utility to identify CMT. Among these biomarkers, NfL demonstrated a particularly strong correlation with the CMTNS, indicating its potential as a reflective marker of disease severity. This finding is consistent with the existing literature and adds to the body of evidence supporting the role of NfL as a biomarker for CMT (3). Furthermore, our study confirmed the well-established correlation between biomarker levels (NfL and GDF15) and patient age, reinforcing the importance of age as a factor in disease progression and biomarker expression (6).

Despite the recognition of NfL and GFAP as markers of axonal damage, our analysis did not reveal a significant difference in levels between the groups of patients with demyelinating and axonal disease. The lack of significant distinction between the groups may be explained by the prolonged course of the disease and the concurrent damage to myelin and axons observed in our cohort. Myelinating cells are known to play a crucial role in supporting axons, in part by supplying metabolic substrates necessary for their energy needs. The specialized structure of myelinated axons, essential for their function in transmitting signals over long distances, makes them especially vulnerable to damage. This vulnerability emphasizes the specific requirements for survival and maintenance of the axon, suggesting that the combined impact of myelin and axonal damage over time could obscure differences in biomarker levels between groups. Three myelin-related protein gene variants—*PMP22*, *MPZ* and *CX32* pathogenic variants, result in a variable degree of both axonal and demyelinating features (17, 18).

GFAP levels have been found to be higher in patients with neurodegenerative disease such as multiple sclerosis and neuromyelitis optica spectrum diseases (13). Astrocytes play a pivotal role in neuronal support and are marked prominently by GFAP. This protein is not only a hallmark of astrocytes in the CNS, but is also present along the peripheral nerve fiber tracks in the peripheral nervous system (PNS). In the PNS, GFAP expression is observed in immature and non-myelinating Schwann cells, with studies indicating that loss of GFAP may lead to poor nerve regeneration after injury. In Alzheimer’s disease, GFAP is linked to the activation of astrocytes surrounding amyloid plaques, a hallmark of AD pathology. Elevated GFAP levels in cerebrospinal fluid (CSF) and plasma correlate with amyloid pathology and are considered a biomarker for disease progression (19, 20).

Despite GFAP and NfL both serving as markers of axonal damage, our findings suggest that GFAP has a comparatively weaker association with disease severity, although it notably increases with disease duration. This distinction highlights the nuanced roles of these biomarkers in chronic neuropathies. The NfL levels are used for multiple sclerosis (MS) subclinical disease activity. They can be monitored regularly in patients with MS to evaluate the change from baseline, relapse risk, and the development of new lesions (21).

GDF15 is a member of the transforming growth factor-beta superfamily has garnered attention for its association with various pathological conditions and the aging process. GDF-15 is up-regulated because it is a systemic biomarker reflecting cellular aging and systemic inflammation (22). GFAP levels have been found to be elevated in a variety of chronic neuropathies, in cases of primary muscular spinal atrophy, and in patients with neurodegenarative diseases (14). Its expression is known to increase with age, suggesting a potential link to age-related diseases. In our study, GDF15 levels have been found to correlate significantly with the age of the group of patients and the duration of CMT disease, indicating its relevance in the progression of hereditary neuropathies. In a study done in 2022 it was found that serum GDF15 was significantly elevated in all subgroups of patients with CMT analyzed and showed that serum GDF15 is highly selective as a diagnostic marker of CMT, they also observed an early elevation of GDF15 in CMT and a significant relationship to the severity of the disease among patients with mildly affected patients, where the CMTNS was lower than 15 (23). Although numerous studies have identified GDF15 as a biomarker linked to peripheral nerve degeneration, its role in muscle metabolism and mass presents a compelling area of investigation (24). The observed inverse relationship between circulating GDF15 levels and skeletal muscle mass and strength underscores a potential connection between GDF15 and muscle atrophy seen in CMT disease (25). This correlation suggests that GDF15 not only serves as a marker of neuropathic processes, but also plays a crucial role in the pathophysiology of muscle degeneration and weakness that typifies CMT. Consequently, GDF15 stands out as a complex biomarker with significant implications for both the diagnostic process and a deeper understanding of the mechanisms of CMT disease. By linking GDF15 to muscle metabolism and mass, we gain valuable insights into the broader systemic effects of CMT on muscular health. GDF15 has been associated with stress responses and inflammation in neurodegenerative conditions. Although its role in CNS demyelinating disorders is less explored compared to neurodegenerative diseases, it may serve as an indicator of cellular stress and systemic inflammation in these disorders (26).

FGF21 plays a pivotal role in the regulation of blood glucose and lipid homeostasis, acting as a key metabolic factor. In particular, its potential protective effect on nerve regeneration has garnered interest, with studies such as Lu et al. (2019) demonstrating FGF21 involvement in mitigating oxidative damage and autophagic cell death during peripheral nerve regeneration (27). This research highlights FGF21 capacity beyond its established metabolic functions, suggesting a therapeutic angle in nerve repair contexts. Furthermore, FGF21 expression has been significantly increased in the muscles of mice with mitochondrial myopathies, where its levels were directly correlated with the presence of COX-negative fibers, a marker associated with disease severity. This observation underscores the relevance of FGF21 in muscular pathology, particularly in conditions marked by compromised mitochondrial function (4, 28). In our investigation, FGF21 levels were elevated in the CMT patient group compared to controls. However, unlike other biomarkers studied, FGF21 did not show significant correlations with disease severity or other parameters examined. This finding is intriguing, given the established role of FGF21 in muscle atrophy and its elevation in mitochondrial disorders and muscle-affected conditions. The lack of significant correlation in our study may reflect the complex interplay of factors in CMT progression and the multifaceted nature of FGF21 actions.

## Conclusion

In our study, we measured the levels of certain plasma biomarkers plasma NfL, GFAP, GDF15, and FGF21 - in individuals diagnosed with CMT disease and compared these levels with a healthy control group. Our findings indicate that people with CMT have significantly higher levels of these biomarkers than those without the disease, pointing to their potential as indicators for CMT diagnosis. The importance of combining biomarker analysis with clinical evaluations is highlighted in our study for a refined understanding of CMT severity. The limitation identified in our research is the restricted sensitivity of CMTNS in capturing the nuances of disease sevierity, underscoring the imperative for the identification and integration of more sensitive biomarkers. Based on previous studies, it is known that CMTNS has not been shown to be sensitive enough to detect meaningful changes within the study period because it showed only a small annual progression in clinical trials, which is why the search for a more sensitive marker for disease progression should be found. Despite its widespread use, CMTNSv2 may have limited sensitivity in detecting subtle early-stage changes, especially in milder forms of the disease. Advances in biomarkers and imaging may complement its application in the future (29, 30). Future directions should consider the integration of plasma biomarker data with advanced diagnostic modalities, including MRI and EMG findings, particularly the motor unit number index (MUNIX), to facilitate a more comprehensive evaluation of patients with CMT. Implementing such a multidimensional approach holds the promise of enhancing the design and precision of clinical trials and longitudinal studies, aimed at developing more precise outcome measures for CMT severity.

In summary, the findings of our study contribute valuable information on the potential utility of plasma levels of NfL, GFAP, GDF15, and FGF21 as biomarkers to assess the severity of CMT. These markers show promise as a reliable outcome measure in the progression of CMT disease.

## Data Availability

The raw data supporting the conclusions of this article will be made available by the authors, without undue reservation.
